# Difficulty and help with activities of daily living among older adults living alone during the COVID-19 pandemic: a multi-country population-based study

**DOI:** 10.1186/s12877-022-02799-w

**Published:** 2022-03-04

**Authors:** Shanquan Chen, Linda A. Jones, Shan Jiang, Huajie Jin, Dong Dong, Xi Chen, Dan Wang, Yun Zhang, Li Xiang, Anna Zhu, Rudolf N. Cardinal

**Affiliations:** 1 Department of Psychiatry, University of Cambridge, Cambridge, CB2 0SZ UK; 2 School of Population and Public Health, University of British Columbia, Vancouver, BC Canada; 3 King’s Health Economics (KHE), Institute of Psychiatry, Psychology & Neuroscience, King’s College London, London, UK; 4 Jockey Club School of Public Health and Primary Care, Chinese University of Hong Kong, Hong Kong, China; 5 Department of Health Policy and Management, School of Public Health, Yale University, New Haven, CT USA; 6 Faculty of Health Sciences, Ontario Tech University, Oshawa, Canada; 7 Institute for Disability and Rehabilitation Research, Ontario Tech University, Oshawa, Canada; 8 Program in Public Health, Renaissance School of Medicine, Stony Brook University, Stony Brook, New York, USA; 9 School of Medicine and Health Management, Tongji Medical College, Huazhong University of Science and Technology, Wuhan, China; 10 Division of Clinical Epidemiology and Aging research, German Cancer Research Center, Heidelberg, Germany; 11 Cambridgeshire and Peterborough NHS Foundation Trust, Cambridge, CB21 5EF UK

**Keywords:** Activities of daily living, Older adults, Live alone, COVID-19, Domiciliary care

## Abstract

**Background:**

Older adults who live alone and have difficulties in activities of daily living (ADLs) may have been more vulnerable during the COVID-19 pandemic. However, little is known about pandemic-related changes in ADL assistance (such as home care, domiciliary care) and its international variation. We examined international patterns and changes in provision of ADL assistance, and related these to country-level measures including national income and health service expenditure.

**Methods:**

We analysed data covering 29 countries from three longitudinal cohort studies (Health and Retirement Study, English Longitudinal Study of Aging, and Survey of Health, Ageing and Retirement in Europe). Eligible people were aged ≥50 years and living alone. Outcomes included ADL difficulty status (assessed via six basic ADLs and five instrumental ADLs) and receipt of ADL assistance. Wealth-related inequality and need-related inequity in ADL assistance were measured using Erreygers’ corrected concentration index (ECI). Correlations were estimated between prevalence/inequality/inequity in ADL assistance and national health-related indicators. We hypothesized these measures would be associated with health system factors such as affordability and availability of ADL assistance, as well as active ageing awareness.

**Results:**

During COVID-19, 18.4% of older adults living alone reported ADL difficulties (ranging from 8.8% in Switzerland to 29.2% in the USA) and 56.8% of those reporting difficulties received ADL assistance (ranging from 38.7% in the UK to 79.8% in Lithuania). Females were more likely to receive ADL assistance than males in 16/29 countries; the sex gap increased further during the pandemic. Wealth-related ECIs indicated socioeconomic equality in ADL assistance within 24/39 countries before the pandemic, and significant favouring of the less wealthy in 18/29 countries during the pandemic. Needs-related ECIs indicated less equity in assistance with ADLs during the pandemic than before. Our hypotheses on the association between ADL provision measures and health system factors were confirmed before COVID-19, but unexpectedly disconfirmed during COVID-19.

**Conclusion:**

This study revealed an unequal (and in some countries, partly needs-mismatched) response from countries to older adults living alone during the COVID-19 pandemic. The findings might inform future research about, and policies for, older adults living alone, particularly regarding social protection responses during crises.

**Supplementary Information:**

The online version contains supplementary material available at 10.1186/s12877-022-02799-w.

## Background

Public health measures such as social isolation and lockdown are effective in containing the spread of COVID-19. However, a resultant reduction in social contact outside the household might marginalize older adults who live alone. These people may be at greater risk for adverse health outcomes than those living with others during the pandemic, because of less direct support and potentially lesser access to essential health and daily services [1–6]. Their vulnerability might be higher if they have difficulties in activities of daily living (ADLs), capabilities associated with the capacity for independent life [1, 7].

Assistance with daily living, such as via domiciliary (home) care, is often essential for adults living alone with ADL difficulties, perhaps especially during a crisis. There is considerable international variation in models of domiciliary care provision, and its funding [8, 9]. Sex differences in disability, measured via ADLs, are well known [10–12]. However, little is known about the matching of ADL assistance (“supply”) to ADL difficulties (“demand”), how this varies across countries and relates to measures of national or individual wealth, or how the provision of ADL assistance has been affected by the COVID-19 pandemic. Such evidence is essential for decision-makers to help understand the situation and address the concerns of people with ADL difficulties. Given the considerable proportion of the elderly population living alone (almost one in four older adults in Europe) [13], evidence is urgently required.

The objective of this study was to estimate the prevalence of older adults having difficulty in ADLs; their receipt of ADL assistance; variations in the provision of ADL assistance in terms of inequality (disparity by wealth) and inequity (disparity by need); international variation in these measures; and the impact of COVID-19 pandemic on these factors. We also examined health system factors, such as affordability and availability of ADL assistance, and active ageing awareness, which might predict these variables.

## Methods

### Study design, setting, and participants

We used publicly available data from three cohort studies collecting relevant self-report data for older adults: 1) the English Longitudinal Study of Ageing (ELSA) in the UK [14], 2) the Survey of Health, Ageing and Retirement in Europe (SHARE), covering 27 countries [15], and 3) the Health and Retirement Study (HRS) in the US [16]. These are large biennial nationally representative individual surveys on people aged ≥50. In brief, HRS is a longitudinal study (launched in 1992) of adults residing in households in the contiguous United States, aged 51–61 at initial study recruitment, and with regular “refreshment” of the cohort with 51–56-year-olds. Its survey topics include demographics, employment/occupation, retirement, disability, earnings/assets, expectations of future events, health status, health care usage, cognitive status, and physical functioning [17]. ELSA, an HRS “sister” study (launched in 2002), is a longitudinal study re-interviewing people ordinarily every 2 years, selected to be representative of those aged 50+ living in private households in England, with regular refreshment of the cohort in each wave to ensure age representation. Among its topics, it covers individual/household demographics, health, social care, social participation, economics/finance, housing, cognitive function, expectations for the future, effort/reward (e.g. volunteering). Additional ad-hoc modules have been added for some waves [18]. SHARE, another HRS “sister” study (launched in 2004), is a similar longitudinal study of adults aged ≥50, systematically sampled initially from 10 European countries but subsequently expanded to more. Its survey data covers health variables, psychological measures, economic variables, and social support variables [19, 20].

Importantly, these studies ran during 2020. Each participant completed a standardised questionnaire, face-to-face (HRS) or via internet/telephone assessments (ELSA, SHARE), described elsewhere [14–16, 21]. Data relevant to the present analysis included sociodemographic characteristics, difficulties in ADLs, and receiving or giving help with ADLs.

We used data from the most two recent survey waves (2020, for all three cohorts, and comparator data from 2018 [ELSA/HRS] or 2017 [SHARE]). Eligible people were those aged ≥50 who lived alone.

### Outcomes of interest

The first outcome was ADL difficulty status, in relation to six basic ADLs (dressing, walking across a room, bathing, eating, getting in and out of bed, toileting) and five instrumental ADLs (preparing a hot meal, shopping for groceries, making phone calls, taking medications, and managing money) [22–24]. Each ADL is assessed similarly, with a question such as “because of a health or memory problem do you have any difficulty with dressing…”, with a yes/no response. During the pandemic, participants were asked about their function since the start of the pandemic. This gave 11 binary indicators of difficulty, one for each ADL. We used two measures of difficulty status: “any ADL difficulty present” (a binary variable) and “extent of ADL difficulty” (sum of ADL scores, range 0–11). Data collected by ELSA in 2020 lacked the questions on ADLs, so those individuals’ difficulty status were taken from their 2018 data. Therefore, we included a sensitivity analysis omitting these data (described below).

The secondary outcome was receipt of assistance with ADLs. The questions used varied slightly across cohort/wave but covered the same domains as difficulty status. We used a binary indicator (yes/no) for receipt of ADL assistance. See Supplementary Methods for detailed questions.

Although both outcomes were calculated from self-reported data, such measures of physical functioning have been evaluated in HRS and HRS-family studies [25].

### Country-level predictor variables

For each country, we also obtained the following measures: (1) gross national income per capita; (2) public expenditure on health as a proportion of GDP; (3) the number of formal long-term care (LTC) workers offering personal care per 100 population aged ≥65; (4) LTC workers offering nursing care per 100 population aged ≥65; (5) informal LTC workforce, reflected by the percentage of the population volunteering to provide ADL help for people outside their own household; and (6) active ageing index (AAI), a United Nations index to monitor national progress on “active ageing”, where higher scores are better [26]. Data on volunteering were obtained from the cohorts. AAI data were from the United Nations [26]. The other five measures are available from the OECD [27] or World Bank [28]. For measures 3 and 4, the cutoff of ≥65y reflects OECD statistics; data were not available for the ≥50y cut-off used in the longitudinal studies above, but the purpose of this measure was not to match the longitudinal studies exactly but to provide a consistent measure, across countries, of the degree of long-term care provided within each country. These variables were measured before the pandemic and their values during the pandemic were predicted from country-specific linear regression (see section below on statistical analysis).

### Ethical approvals

The data were publicly available. The use of public secondary de-identified data made the present study exempt from institutional review board review. Participants in the original studies gave informed consent and each study was approved by a relevant ethics body: for ELSA, the UK Health Research Authority South Central Berkshire Research Ethics Committee [14, 18]; for HRS, the University of Michigan Institutional Review Board [29]; for SHARE, the Ethics Council of the Max Planck Society plus ethics committees in participating countries [30].

### Statistical analysis and comparisons

Data were analysed by country. Survey weighting per wave was used to account for sampling design (including the unequal probability of selection, clustering, and stratification) and study attrition.

To estimate sex disparity, and any mediating effect of the COVID-19 pandemic on sex disparity, among those receiving ADL assistance, we fitted country-specific weighted logistic regression models. The dependent variable was receipt of ADL assistance. Predictors were: age (continuous), sex (female/male), wealth (continuous; see Supplementary Methods), the extent of ADL difficulty (continuous), pandemic (yes = data collected in 2020; no = earlier), and the sex × pandemic interaction. We hypothesized that there would be no sex disparity in receipt of ADL assistance, and no influence from COVID-19 on sex disparity.

To measure inequality in receipt of ADL assistance over the socioeconomic distribution, we calculated the Erreygers’ corrected concentration index (ECI) for each survey wave by country, taking receipt of ADL assistance as the outcome variable and wealth as the reference variable, following World Bank guidelines [31]. This index is commonly used to measure socioeconomic inequality in the health sector. The theoretical ECI range is −1 to 1. If the receipt of assistance is not correlated with wealth, the ECI is zero (no socioeconomic inequality). A positive ECI indicates a disproportionate receipt of ADL assistance among richer individuals (“pro-rich”), and conversely for negative values (“pro-poor”). Additionally, to understand whether people with higher needs (reflected by ADL difficulty) received more ADL assistance, we calculated the need-related ECI, using ADL difficulty as the reference variable instead. Here, positive values indicate greater receipt of help amongst those with greatest need. To eliminate the confounders, the wealth-related ECI was standardised for age, sex, and ADL difficulty; the need-related ECI was standardised for age, sex, and wealth. See Supplementary Methods for formulae. We hypothesized that standardised wealth-related ECI was zero (equal receipt of help regardless of wealth), and that the standardised need-related ECI was positive (“pro-difficulty”, i.e. help going more to those with greater need). Note that a zero wealth-related ECI suggests equality (resources allocated equally regardless of wealth) but not necessarily equity (for which greater resource allocation to those with less wealth might be expected).

We related receipt of ADL assistance, wealth-related ECI, and need-related ECI to six measures for each country (described above) relating to the affordability and availability of ADLs assistance, as well as active ageing awareness—collectively referred to as welfare levels. e adopted Kendall’s rank correlation coefficient (Kendall’s tau) to measure the strength and direction of the association between each of these variables and the proportion receiving ADL assistance, and wealth- or need-related ECI. We calculated R^2^ by linear regression to establish the proportion of variance explained by each predictor. We hypothesised that increasing welfare support in a country would be associated with a greater receipt of ADL assistance and greater equality/equity in such receipt.

Missing data were imputed. For wealth, we used multiple imputations with chained equations and generated five imputed data sets to reduce bias and maintain power [32]. For “health system” level measures, we fitted country-specific linear regression using data from 2010 to 2019 and imputed the missing values using regression predictions.

We performed a structural sensitivity analysis. To eliminate the possible influence of different questionnaires used by the three cohorts, we repeated the analysis including only SHARE data.

Analyses used R version 3.6.0. We report two-tailed *P* values and 95% confidence intervals (CIs) throughout. *P* < .05 was considered statistically significant. Results are reported following the STROBE checklist for cohort studies.

## Results

A total of 15,648 individuals were included in the survey waves after the COVID-19 outbreak, and 28,137 individuals before COVID-19 as a comparison group. More than 70% of older adults living alone were female (72.7% during the pandemic, and 70.7% before). Before the pandemic, across all countries included, 27% of older adults living alone reported ADL difficulties (ranging from 12.8% in Switzerland to 35.6% in Portugal) and 51.5% of those reporting difficulties received help with ADLs (ranging from 6.2% in Romania to 85.1% in the Netherlands). During the pandemic, 19.6% of older adults living alone reported ADL difficulties (ranging from 8.8% in Switzerland to 28.5% in the USA) and 56.3% of those reporting difficulties received help with ADLs (ranging from 25.5% in Israel to 77.1% in Greece); see Table 1.

**Table 1:** Basic description of ADL difficulties and support across countries before and during the COVID-19 pandemic. Categorical variables are reported as number (percentage) and continuous variables as mean (standard error). The results for all countries are not weighted because of the inconsistency (in frequency weights or sampling weights) of the weight value for individuals within each country

Country	Before the COVID-19 pandemic						During the COVID-19 pandemic					
	*N*	Age Mean (SE)	Female *n* (%)	Report any difficulties with ADLs			*N*	Age Mean (SE)	Female *n* (%)	Report any difficulties with ADLs		
				*n* (%)	Extend of ADL difficulties Mean (SE)	Received assistance with ADLs; *n* (%)				*n* (%)	Extend of ADL difficulties Mean (SE)	Received assistance with ADLs; *n* (%)
Belgium	1299	71.1 (0.49)	861 (64.5%)	395 (27.9%)	3.3 (0.15)	257 (65.4%)	1127	71.4 (0.58)	777 (65.6%)	256 (22.4%)	2.5 (0.14)	166 (61.9%)
Bulgaria	482	69.9 (0.51)	363 (71%)	114 (23.8%)	3 (0.24)	26 (21.5%)	229	72.1 (0.76)	175 (71.7%)	46 (19.4%)	2.6 (0.3)	27 (56.7%)
Croatia	344	70.8 (0.62)	251 (71.3%)	67 (21.3%)	3.2 (0.34)	13 (19.6%)	338	71.5 (0.64)	244 (68.4%)	46 (14%)	2.9 (0.34)	33 (69.8%)
Cyprus	224	74.3 (0.85)	184 (77.2%)	73 (26.2%)	4.2 (0.43)	43 (57.5%)	149	76.3 (0.96)	122 (79.2%)	34 (18.4%)	2.4 (0.47)	24 (65.1%)
Czech Republic	1125	71 (0.76)	899 (78%)	270 (20.7%)	3.3 (0.23)	140 (55.7%)	789	71.9 (0.79)	647 (79.1%)	106 (12.3%)	2.2 (0.21)	57 (48.6%)
Denmark	780	71.1 (0.44)	500 (61.4%)	165 (20.2%)	3.3 (0.22)	116 (69.2%)	495	72.4 (0.59)	322 (59.5%)	59 (11.9%)	2.1 (0.25)	24 (39.1%)
Estonia	1582	70.1 (0.32)	1228 (72%)	427 (23.1%)	3.1 (0.12)	66 (15%)	1545	71 (0.34)	1233 (72.7%)	288 (17.6%)	2.5 (0.13)	160 (52.7%)
Finland	437	70 (0.84)	269 (61.6%)	79 (16.9%)	2.3 (0.29)	28 (35.4%)	313	71 (0.95)	215 (65.2%)	50 (14.6%)	1.7 (0.18)	25 (57%)
France	984	72.6 (0.53)	702 (67.8%)	247 (23.5%)	3.4 (0.2)	161 (64.7%)	692	72.2 (0.7)	502 (64.4%)	98 (13.8%)	2.5 (0.24)	61 (58.6%)
Germany	829	71.2 (0.45)	516 (61.7%)	190 (22.7%)	3 (0.2)	108 (57.8%)	688	71.8 (0.48)	444 (62.4%)	105 (15.2%)	2.3 (0.18)	74 (69.1%)
Greece	697	72.8 (0.58)	519 (72.9%)	140 (20%)	3.1 (0.26)	98 (71.1%)	796	73.5 (0.5)	593 (73.1%)	90 (15.5%)	2.8 (0.27)	68 (77.1%)
Hungary	372	71.4 (0.82)	294 (76%)	89 (22.3%)	2.7 (0.35)	27 (35.6%)	233	73.3 (0.91)	188 (76.4%)	30 (12.4%)	3.1 (0.99)	19 (71.6%)
Israel	432	67.6 (1.79)	329 (65.9%)	167 (25.3%)	3.7 (0.29)	100 (65.4%)	336	70.2 (1.41)	272 (74.3%)	79 (27.9%)	2.6 (0.27)	38 (25.5%)
Italy	703	72.2 (0.68)	499 (69.4%)	182 (24.7%)	4.1 (0.26)	105 (60.6%)	628	72.1 (0.78)	459 (68.4%)	109 (17%)	3.1 (0.28)	72 (69.1%)
Latvia	582	70 (0.46)	450 (71.6%)	130 (20.5%)	3.1 (0.23)	32 (24.3%)	319	71.8 (0.64)	254 (73.1%)	61 (17.9%)	3.2 (0.36)	28 (44.6%)
Lithuania	651	70.3 (0.44)	517 (76.5%)	149 (22.3%)	3.3 (0.22)	20 (13%)	406	71.9 (0.58)	331 (79.6%)	69 (14.4%)	3.2 (0.34)	56 (79.5%)
Luxembourg	224	73.8 (0.82)	141 (63.8%)	45 (20.6%)	3.5 (0.41)	26 (59.5%)	176	75.2 (1.02)	116 (68.2%)	17 (13.3%)	3.2 (0.76)	12 (67.4%)
Malta	172	71.4 (0.87)	127 (69.2%)	34 (19.1%)	3.5 (0.51)	7 (19.9%)	121	73.6 (1.08)	88 (67%)	22 (17.9%)	2.8 (0.56)	10 (48.8%)
Netherlands	842	69.8 (0.32)	530 (62.9%)	121 (14.4%)	2.4 (0.19)	103 (85.1%)	214	70.5 (1.43)	141 (61.6%)	13 (9.7%)	2.4 (0.49)	7 (48.7%)
Poland	663	69.6 (0.48)	450 (66.8%)	157 (23.4%)	2.9 (0.21)	64 (38.3%)	451	71.2 (0.58)	315 (69.3%)	91 (20.4%)	2.9 (0.31)	67 (74.8%)
Portugal	186	72.4 (2.61)	133 (67.4%)	61 (35.6%)	3.9 (0.59)	18 (17.1%)	163	74.9 (2.1)	124 (79.1%)	34 (17.7%)	2.4 (0.3)	18 (66.1%)
Romania	320	72 (0.75)	229 (70%)	104 (34.3%)	3 (0.29)	7 (6.2%)	232	71.2 (0.87)	174 (71.1%)	63 (27%)	3.5 (0.34)	47 (75.9%)
Slovakia	298	68.3 (0.75)	192 (67.3%)	35 (17.1%)	2.4 (0.29)	7 (11.9%)	176	69.4 (0.82)	118 (66%)	16 (10.7%)	2.9 (0.55)	9 (54.7%)
Slovenia	675	72.4 (0.57)	511 (67.9%)	161 (20.5%)	3.6 (0.25)	26 (17.4%)	648	74 (0.57)	499 (70.8%)	104 (15.6%)	3.2 (0.28)	59 (54.4%)
Spain	841	73.9 (0.73)	574 (66.5%)	230 (25.9%)	4.3 (0.28)	126 (61.7%)	419	76.3 (0.92)	299 (72%)	83 (19.7%)	3.8 (0.34)	45 (50.7%)
Sweden	862	71.7 (0.54)	575 (62.8%)	173 (16.1%)	3 (0.22)	101 (54.1%)	413	73.2 (0.86)	267 (57.1%)	50 (12%)	2 (0.22)	36 (68.4%)
Switzerland	619	71.6 (0.57)	426 (64.8%)	86 (12.8%)	2.6 (0.27)	56 (63.2%)	541	73.2 (0.81)	374 (63.6%)	47 (8.8%)	1.5 (0.17)	34 (76%)
UK	2340	67 (0.29)	1591 (62.3%)	656 (27.2%)	3.2 (0.14)	371 (61.2%)	1541	70.8 (0.41)	1039 (60.7%)	319 (25.2%)	2.7 (0.14)	108 (34.4%)
USA	7544	69 (0.14)	5257 (69.7%)	2622 (34.8%)	3.4 (0.05)	1515 (57.8%)	1470	68.8 (0.37)	1046 (64.6%)	439 (28.5%)	3.3 (0.18)	196 (46.5%)
All countries	28,137	71.8 (0.06)	19,880(70.7%)	7608 (27.0%)	3.35 (0.03)	3920 (51.5%)	15,648	74.2 (0.08)	11,378 (72.7%)	2824 (19.6%)	2.78 (0.05)	1580 (56.3%)

**Fig. 1:**
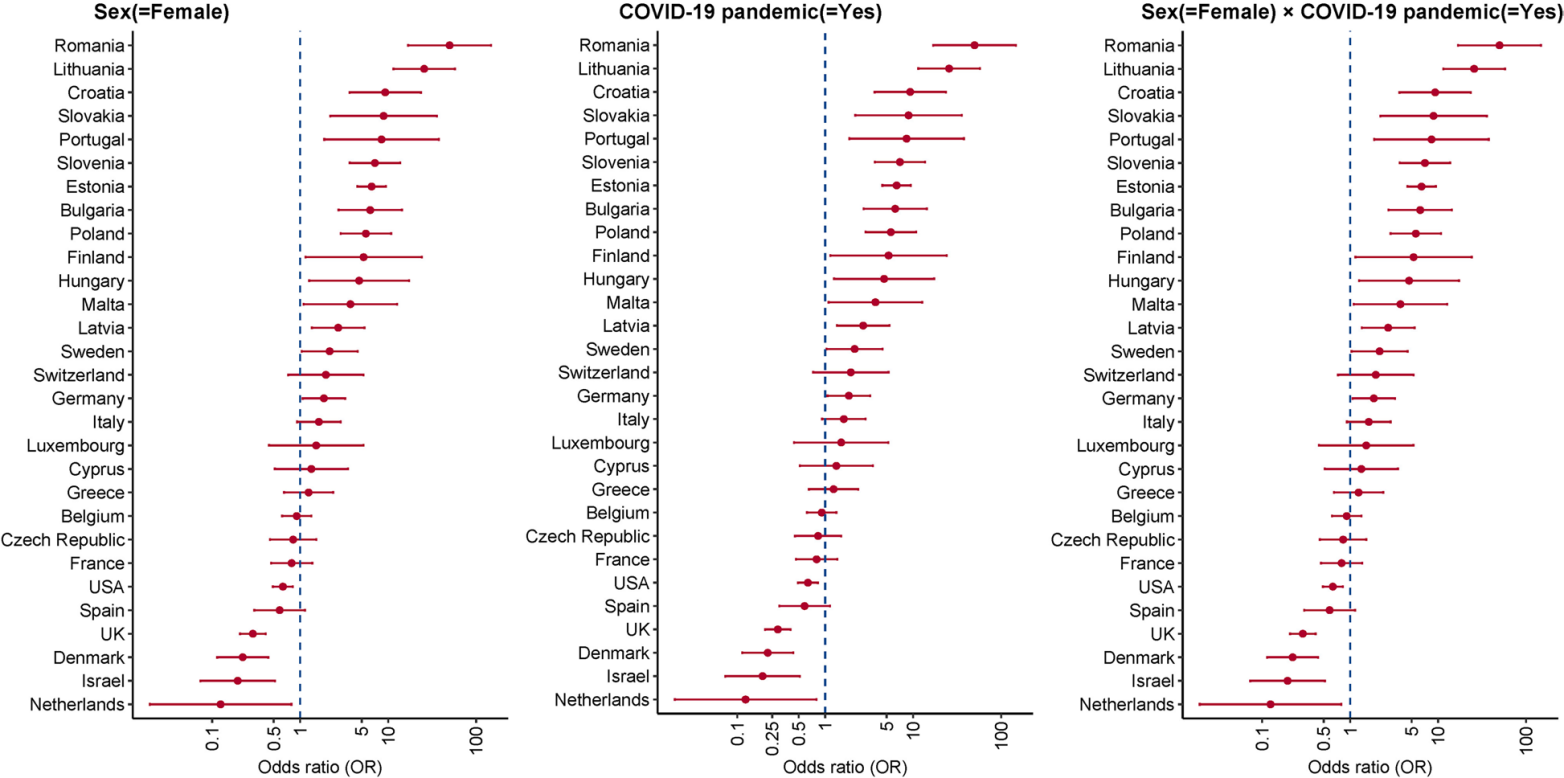
Sex disparity and influence of COVID-19 pandemic on the receipt of assistance with ADLs. Effects are shown from country-specific weighted logistic regression models, with receipt of ADL assistance as the dependent variable, and age (continuous variable), sex (female versus male), wealth (continuous variable), the extent of ADL difficulty (continuous variable), year (2020 survey waves [COVID-19 pandemic] versus the previous survey wave), and the interaction term of sex and pandemic, as independent variables

Figure 1 shows the sex disparity and influence of the pandemic on receipt of ADL assistance. After controlling for age, wealth status, and extent of difficulty, females were significantly more likely to receive ADL assistance than males in 16/29 countries (Fig. 1A). The probability for females to receive ADL assistance increased significantly within 13/29 countries during the pandemic (Fig. 1C). Figure 1B also shows that during the pandemic, the probability of receiving ADL help increased significantly within 15/29 countries (Romania, Lithuania, Croatia, Slovakia, Portugal, Slovenia, Estonia, Bulgaria, Poland, Finland, Hungary, Malta, Latvia, Sweden, and Germany), and decreased significantly within 5/29 countries (the USA, UK, Denmark, Israel, and the Netherlands).

**Fig. 2:**
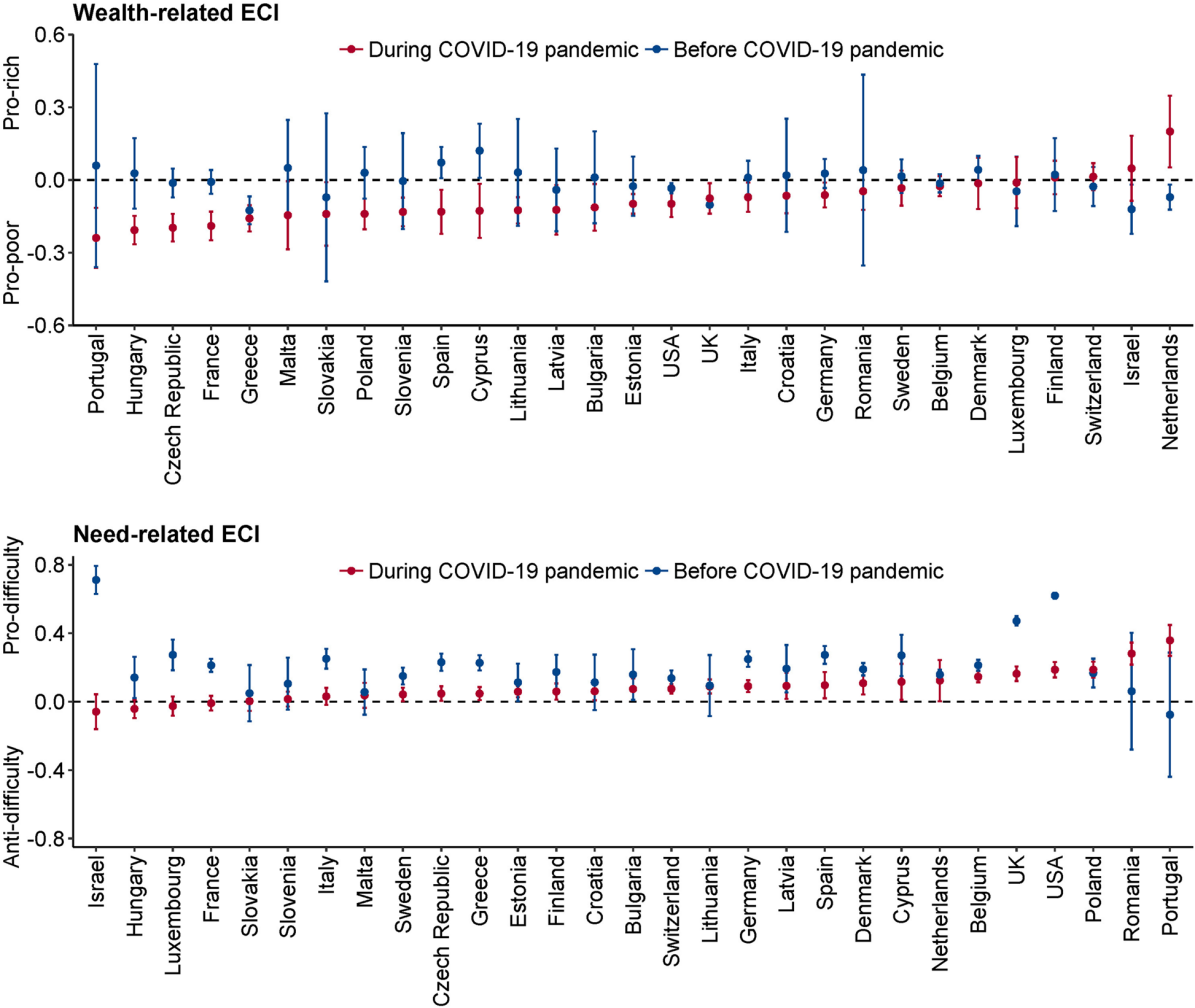
Wealth-related and need-related Erreygers’ corrected concentration index (ECI) for receipt of assistance with ADLs. **A** Wealth-related Erreygers’ concentration index was standardised for age, sex, and the extent of ADL difficulty. **B** Need-related Erreygers’ concentration index, standardised for age, sex, and wealth status. A positive ECI indicates a concentration of ADL assistance towards wealthier individuals (wealth-related ECI) or people with greater ADL difficulties (need-related ECI)

Standardized wealth-related and need-related ECIs for receipt of ADL assistance are shown in Fig. 2. Before COVID-19, the wealth-related ECIs were not significantly different from zero and indicated socioeconomic equality (in this respect) within 22/29 countries, but during the pandemic, these values became significantly negative, indicating “pro-poor” receipt of ADL assistance, within 19/29 countries (Fig. 2A). Figure 2B shows that during the pandemic the need-related ECIs reduced in almost all countries (indicating that those with greater difficulty received a lesser proportion of total support than before), but remained positive, still indicating a “pro-difficulty” distribution of ADL support.

**Fig. 3:**
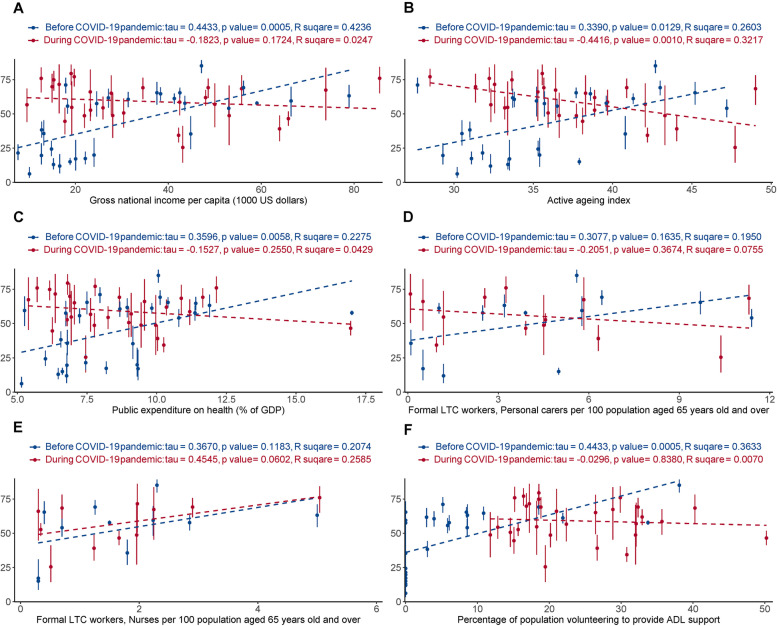
Percentage of people receiving assistance with ADLs (y axis) versus national-level health system factors (x axis). The blue (before pandemic) and red (during pandemic) fit lines intersect mainly in the middle of each plot, indicating the changes are driven by countries with both low and high “welfare” indicators (*x* axes). Note that the abscissa (x axis coordinate) for a given country may differ before versus after the pandemic (see Methods)

Before COVID-19, the proportion of people receiving ADL assistance was positively associated with gross nation income per capita (τ = 0.44, *p* = 0.0005, R^2^ = 0.42), AAI (τ = 0.34, *p* = 0.0129, R^2^ = 0.26), public expenditure on health (τ = 0.36, *p* = 0.0058, R^2^ = 0.23), and percentage of the population volunteering to provide ADL support (τ = 0.44, *p* = 0.0005, R^2^ = 0.36). After the COVID-19 pandemic began, it was negatively associated with AAI (τ = − 0.44, *p* = 0.001, R^2^ = 0.32), and not other variables (Fig. 3).

**Fig. 4:**
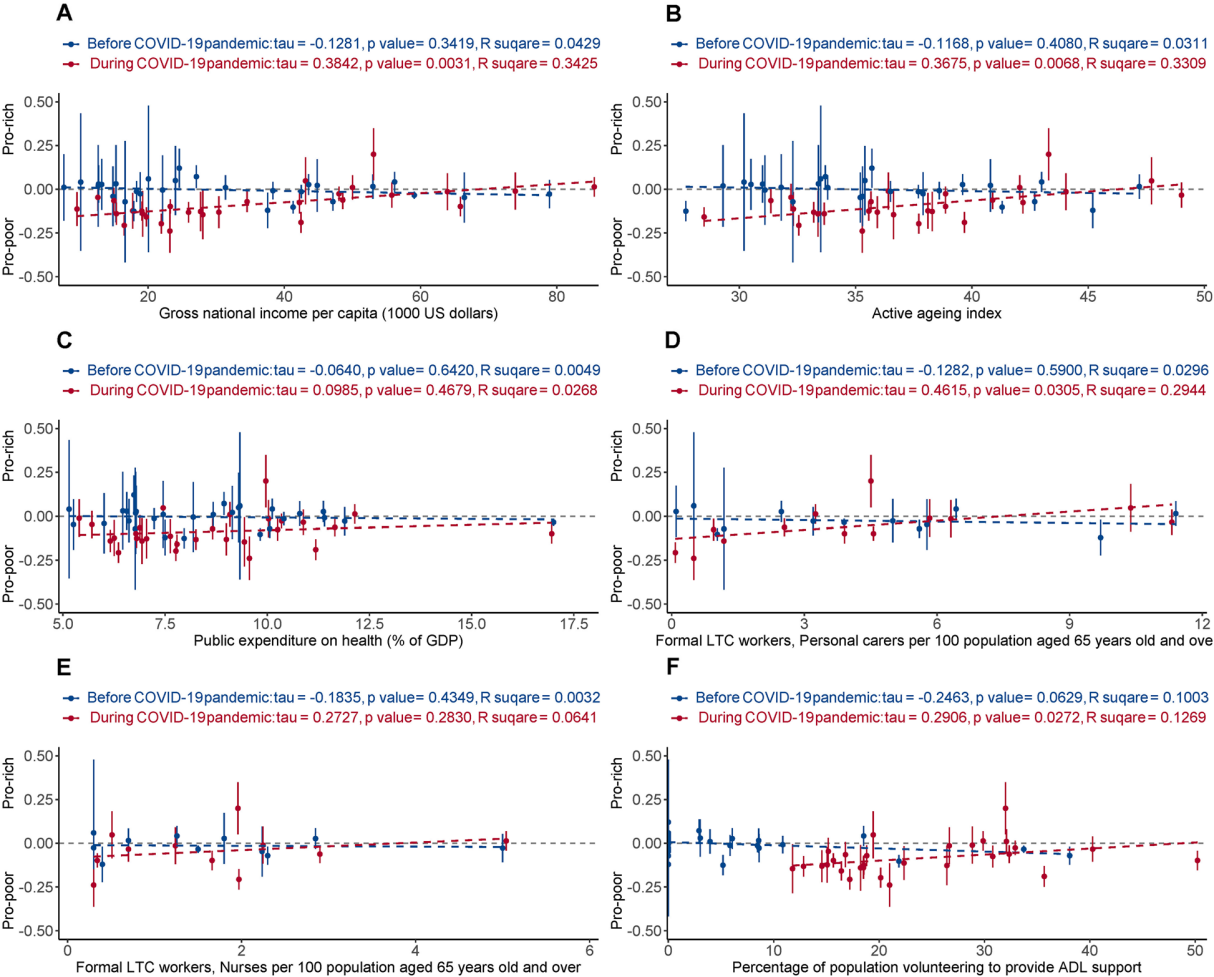
Wealth equality/inequality in receiving assistance with ADLs (ordinate, y axis) versus national-level health system factors (abscissa, x axis). The wealth-related Erreygers’ concentration index (ECI) was standardised for age, sex, and the extent of ADL difficulties. The blue and red dashed lines represent the fit of wealth-related ECI and the national-level measure. The intersections of the blue and red lines are primarily towards the right, indicating that the changes came mostly from “low welfare” countries. Note that the abscissa (x axis coordinate) for a given country may differ before versus after the pandemic (see Methods)

Before COVID-19, none of the six country-level measures showed a significant association with wealth-related ECI. During COVID-19, wealth-related ECI was positively associated with gross national income per capital (τ = 0.38, *p* = 0.0031, R^2^ = 0.34), AAI (τ = 0.37, *p* = 0.0068, R^2^ = 0.33), the LTC workforce for personal care (τ = 0.46, *p* = 0.0305, R^2^ = 0.29), and volunteering (τ = 0.29, *p* = 0.027, R^2^ = 0.13) (Fig. 4).

**Fig. 5:**
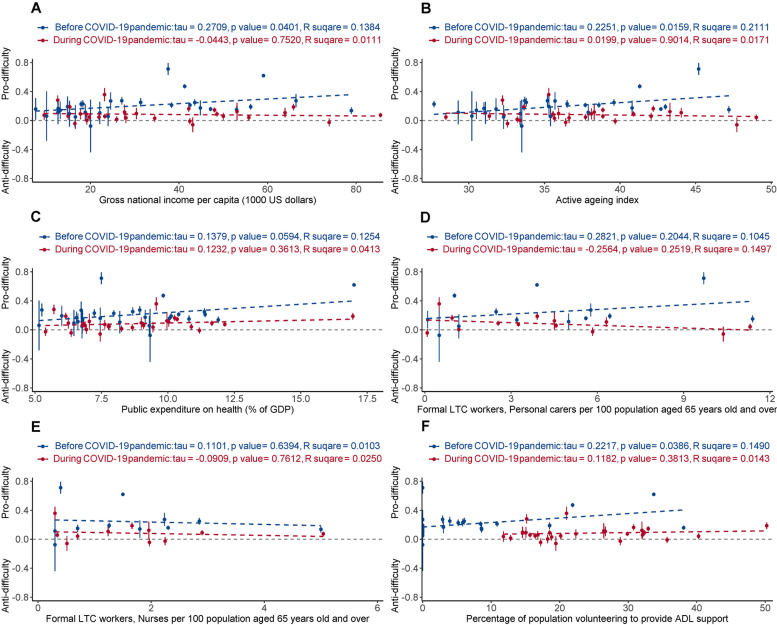
Needs-based equity/inequity in receiving assistance with ADLs (y axis) versus national-level health system factors (x axis). The need-related Erreygers’ concentration index (ECI) was standardised for age, sex, and wealth status. The blue and red dashed lines are show the linear fit. The intersections of blue and red line are mainly towards the left, indicating that the changes were driven primarily by “high welfare” countries. Note that the abscissa (x axis coordinate) for a given country may differ before versus after the pandemic (see Methods)

Before COVID-19, the need-related ECI was positively associated with gross national income per capita (τ = 0.27, *p* = 0.040, R^2^ = 0.14), AAI (τ = 0.23, *p* = 0.0159, R^2^ = 0.21), and volunteering (τ = 0.22, *p* = 0.0386, R^2^ = 0.15). In contrast, during COVID-19, none of the six country-level resource measures were correlated with need-related ECI (Fig. 5).

The pandemic-related changes (Figs. 3, 4, 5) were statistically significant, as indicated by supplementary analyses adding “before versus after COVID-19” as an explicit predictor (Supplementary Table 1).

These results were consistent in sensitivity analyses (Supplementary Figs. 1, 2, 3).

## Discussion

### Statement of principal findings

To our knowledge, this is the first study to assess difficulty and help with ADLs internationally among older adults living alone during COVID-19. During the pandemic, fewer people reported problems with ADLs, and a higher proportion of those reporting ADL difficulties received assistance than before, but still about two-fifths of those reporting ADL difficulties did not receive any assistance. Females were more likely to receive ADL assistance in those countries for which there was a sex effect (controlling for self-reported need), and in many countries this sex effect increased during COVID-19. The distribution of ADL assistance shifted towards less wealthy people but also towards people with slightly fewer needs. Provision of ADL assistance was positively associated with national welfare indicators before COVID-19, and likewise for need-based equity/inequity in ADL assistance, but these associations disappeared. Wealth-based equality/inequality in assistance was not associated with welfare indicators before COVID-19, but this association became positive. Overall, this study revealed a variability in countries’ ability to provide support for older adults living alone during the pandemic.

### Interpretation

Compared with the pre-pandemic period, fewer older adults living alone reported ADL difficulties during the pandemic (Table 1). This might be because some older adults with ADL difficulties were joined by their relatives or friends, putting them outside the scope of the relevant surveys. Another distinct possibility is that those with greater needs were those at highest risk of death in the pandemic [33], distorting the distribution of needs in the surviving population. For the rest of the people who were still living alone during the COVID-19 pandemic, our results are compatible with a response from the local care systems, because a higher proportion of people received ADL assistance than before the pandemic (Table 1 and Fig. 1), although it should be noted that again a possibility is of delivery of care to a population within which a subgroup of vulnerable people with the greatest need had died. Both of these effects suggest a positive response to older adults living alone during the COVID-19 pandemic. However, more concerningly, about two-fifths of those reporting ADL difficulties did not receive any assistance during the pandemic. This gap was increased to more than 50% in some countries, namely the Czech Republic, Denmark, Israel, Latvia, Malta, Netherlands, the UK, and the USA.

Females were more likely to receive ADL assistance than males in 16/29 countries, even after controlling for age, wealth, and the extent of difficulty. There have been similar finding from Japan [34] and the USA [35, 36] indicating that females are more likely to receive social support than males. One possible reason might be that females are, on average, more active in neighbourhood social networks and are more likely to ask for help when in need [24, 37]. This might also explain why, when needs increased during the pandemic, the probability of receiving ADL assistance increased significantly more for females than males in countries where there was a change (Fig. 1C). The sex disparity in receiving assistance with ADLs might result in males living alone being more likely to experience unmet health needs than females [34, 38–40]. For a comprehensive social support response or intervention during crises like COVID-19, this sex disparity deserves to be noted.

Our hypotheses regarding socioeconomic equality and equity in receiving ADL assistance were confirmed for the period before the pandemic, but disconfirmed during the pandemic. During the pandemic, ADL assistance was provided more to those of lower wealth status (in 19/29 countries) (Fig. 2A). However, where changes were observed, the distribution of ADL assistance shifted towards those with lower ADL difficulties during the pandemic (Fig. 2B). This might indicate a disruption of social support and a lack of matching of provision to need during the pandemic (though disproportionate death amongst those with greater need may have also played a role, as above). This finding was widespread across countries.

Figures 3, 4, 5 provide insight into factors that might explain part of the international variation in care delivery. As might be expected, some cross-country variations were explained by “welfare provision” factors, but their explanatory power was affected by the pandemic. Before COVID-19, higher levels of welfare provision in a country were associated with a higher proportion of those receiving ADL assistance, and greater needs-based allocation of ADL assistance, and were not associated with recipient wealth. Unexpectedly, these associations shifted in the opposite direction during the COVID-19 pandemic. The degree of change may relate to the baseline level of the welfare indicators. For example, the countries where the proportion of people receiving ADL assistance increased most during the pandemic tended to be those countries with relatively low levels of welfare indicators (e.g. Croatia, Hungary, Poland, Romania, Slovenia, Slovakia, Portugal, Lithuania, and Bulgaria) (Fig. 3). By contrast, countries that unexpectedly had a decrease in the proportion receiving ADL assistance were those countries with relatively high levels of welfare provision (e.g. Netherlands, Denmark, the USA, Israel, and the UK) (Fig. 3) – despite these being countries across which COVID-19 death rates have varied widely [41]. These unexpected findings suggest a possible weakness in “high-welfare” countries in caring for older adults living alone with ADL difficulties during crises like COVID-19.

Do these findings provide practical suggestions for countries providing care to older adults living alone during a crisis? Among the six country-level factors examined, the informal LTC workforce (volunteers) is notable as a factor that might be changed rapidly in the short term, and it did increase during COVID-19, particularly in countries with a relatively low level of welfare provision (Supplementary Fig. 4). This might be why these countries simultaneously had an increase in the proportion of those receiving ADL assistance (Supplementary Fig. 4). This causality remains to be verified in future studies, but would suggest that encouraging volunteering (and systematically allocating volunteers) is a feasible and flexible way to help older adults living alone, including during crises. Figures 3(D-F) and 5(D-F) also show that both the proportion receiving ADL assistance, and needs-based equity, were (for some measures) significantly and positively associated with informal LTC workforces (but not with formal LTC workforces) before COVID-19 than during the pandemic, which might suggest barriers to accessing formal carers before and during the pandemic and/or non-optimal allocation of volunteers during the pandemic. Additionally, Figs. 4-5 show that during COVID-19, changes in “wealth-associated” allocation of ADL assistance were mainly driven by countries with a relatively low level of welfare provision, whereas changes in “needs-associated” provision were driven more by those with high levels of welfare provision. A challenge for all countries is to ensure equitable allocation of resources to needs, if needs cannot be met in full. Finally, the absolute level of support provision across survey waves indicates that a significant fraction of the population reported difficulties with ADLs but was not in receipt of support. This apparent level of unmet need is of some concern, though the true picture might not be as stark as these figures suggest because use of binary questions about ADL difficulty did not allow us to measure the extent to which support was required, e.g., distinguishing mild impairment from severe functional impairment. We discuss this point further with respect to study limitations.

### Implications for future research

It has been widely reported that lower socioeconomic status is linked to worse health status [42]. Therefore, lower socioeconomic status has been used as a common measure to identify those people in need [43]. However, this approach might not result in the most equitable response during crises, as identified in our study. More precise measures are required to identify people most in need and creative interventions may be needed to help older adults to access the services they need during a crisis. This may include informal carers, as discussed above. Although some studies have suggested that smart software “apps” could facilitate older people receiving home care [44] and that team-based primary care could reduce the sex disparity in disabled people receiving services [45], none of these studies examined people living alone with ADL support needs or in the context of crises such as COVID-19.

The challenges associated with living alone have long received attention, and many countries have worked to develop active-ageing societies [46]. Positively, higher “welfare” indices were in general associated with a higher proportion of people receiving ADL assistance when needed (Fig. 3), and some countries increased support during COVID-19 (Fig. 1B); less positively, this study suggests high levels of unmet need even in high-income countries (e.g. Table 1) and in some cases a proportional decline in support during the pandemic (Fig. 1B). Future studies might explore the impact of “health” versus “social” care and whether these are treated as distinct, the extent of attention received by older adults living alone, sex disparity, and the culture or the circumstances that could affect these.

## Strengths and limitations

A strength of this study is that the representative cohort data enabled the comparison of support for older adults living alone, before and during the pandemic, in relation to their needs. The multi-country comparison against country-level health system factors allowed comparison of countries’ response to caring for this group. Considering needs-based inequity as well as socioeconomic inequality allowed a more nuanced and practical assessment of care delivery.

Our study also has a number of limitations. First, it would be useful to further explore whether those reporting ADL difficulties received quantitatively more help during the pandemic; this was considered unfeasible due to the differences between questions used among countries and between survey waves. Therefore, we decided to use a simple binary indicator to reflect the receipt of ADL assistance for practical purposes. Likewise, the use of binary questions about ADL difficulty did not allow differentiation of mild from severe impairment within a given ADL domain. Second, it is relevant to ask whether there is ethnic disparity in people receiving ADL assistance, but ethnicity information was not collected by SHARE. Although ELSA and HRS collected relevant information, small subgroups hampered analysis. Third, a positive need-related ECI indicates that ADL assistance was given proportionally more to those with higher ADL difficulties, but is this is not categorical proof of equity (for example, provision of support to everyone in need might be considered ideal yet score ~ 0, whereas provision of support only to those in maximal need might score ~ 1 yet leave many people without desperately needed support). In this study, we took the value before the pandemic as a reference to examine how equity changed. Fourth, volunteering is a potential modifiable factor identified in this study, but its influence could be underestimated, as the percentage of the population volunteering to provide ADL assistance was calculated from cohorts surveying people aged ≥50, and not all populations. Fifth, individuals’ difficulty status in the UK was matched to their data collected in 2018, not 2020; this could bias UK results, but is unlikely to have influenced our general conclusions, borne out by consistent sensitivity analyses. Sixth, other factors, such as education or other social determinants of health, might mediate the relationship between ADL impairment and seeking or obtaining assistance but were not examined in the current study. Seventh, we examined the situation country by country, but only limited national-level factors were explored. In addition, for many countries only limited data on formal long-term care workforce were available.

## Conclusion

There was an unequal international response in the provision of ADL support to older adults living alone during the COVID-19 pandemic, with evidence suggesting under-provision of support to many. The proportion of the elderly population living alone is expected to increase, as our societies face an ageing trend, and countries should consider their future preparedness to provide support to vulnerable individuals during times of crisis as well as normality.

## Supplementary Information

**Additional file 1: Supplementary Methods.** Questions used in data collection, formula to calculate the (Erreygers’ corrected) concentration index, and measures of wealth status. **Supplementary Table 1.** Testing the influence of the COVID-19 pandemic on the association between national level factors and outcomes of interest. **Supplementary Figures 1-3.** Sensitivity analyses, with conventions as for Figs. 3, 4, 5, respectively. **Supplementary Figure 4.** Change in the percentage of population volunteering to provide ADL support against change in the percentage of people receiving assistance with ADLs.

## Data Availability

The individual-level datasets analysed during the current study are publicly available in the English Longitudinal Study of Ageing, https://www.elsa-project.ac.uk/accessing-elsa-data; Survey of Health, Ageing and Retirement in Europe, http://www.share-project.org/data-access.html; and Health and Retirement Study, https://hrsdata.isr.umich.edu/data-products/rand. The country-level datasets analysed during the current study are extracted from United Nations report, https://unece.org/DAM/pau/age/Active_Ageing_Index/ECE-WG-33.pdf; or publicly available in OECD data, https://stats.oecd.org/; and World Bank Open Data, https://data.worldbank.org/.

## Abbreviations

AAI: Active ageing index
ADL: Activities of daily living
ECI: Erreygers’ corrected concentration index
ELSA: English Longitudinal Study of Ageing
GDP: Gross domestic product
HRS: Health and Retirement Study
LTC: Long-term care
OECD: Organisation for Economic Co-operation and Development
SHARE: Survey of Health, Ageing and Retirement in Europe
UK: United Kingdom
USA: United States of America
