# Supplementary material for: Difficulty and help with activities of daily living among older adults living alone during the COVID-19 pandemic: a multi-country population-based study
Source: BMC Geriatr. 2022 Mar 4;22:181. doi: 10.1186/s12877-022-02799-w (PMC8894568; doi:10.1186/s12877-022-02799-w)

**SUPPLEMENTARY MATERIAL**

***Difficulty and help with activities of daily living among older adults living alone during the COVID-19 pandemic: a multi-country population-based study***

***SUPPLEMENTARY METHODS***

**Questions used in data collection on receipt of assistance with activities of daily living (ADLs)**

- In 2020 survey waves, receipt of ADL assistance was primarily assessed by broad questions.
  - In ELSA, the question was “Over the past month, have you received care at home?”, with possible answers “Yes, formal (paid, provided from an agency)”, “Yes, informal (friend or relative)” or “no”.
  - In SHARE, the question was “Since the outbreak of coronavirus, were you helped by others from outside of home to obtain necessities, e.g. food, medications or emergency household repairs?”, with answers “yes” or “no”.
  - In HRS, the questions were “Because of the coronavirus pandemic, did anyone living outside your household, such as a parent, adult child, other relatives, or friends, help you with money or by paying bills?” and “Because of the coronavirus pandemic, did anyone living outside your household, such as a parent, adult child, other relatives, or friends, help you with shopping for groceries, errands, rides, or chores?”, with answer “yes” or “no”.
- Note that for data collection during the pandemic, participants were asked about their function since the start of the pandemic.
- In 2017/2018 survey waves, receipt of ADL assistance was mainly assessed by ADL-specific questions.
  - In ELSA, help received for each ADLs (except preparing a hot meal) was assessed with a specific question such as “Have you received help from anyone with dressing, including putting on shoes and socks, in the last month?”, with two possible responses: yes and no. Help with meals was assessed with the question “Had your family, friend or neighbour bring you ready prepared meals?” with two possible responses: yes or no.
  - In SHARE, ADL help was assessed with the questions: “Thinking about the activities that you have problems with, does anyone ever help you with these activities?” with two possible responses (yes or no); “During the last twelve months, did you receive in your own home any professional or paid services listed on this card due to a physical, mental, emotional or memory problem?” (yes or no); “Thinking about the last twelve months, has any family member from outside the household, any friend or neighbour given you any kind of help listed on this card?” (yes or no); and “Is there any other family member from outside the household, friend or neighbour who has given you personal care or practical household help?” (yes or no). Services listed included personal care (e.g. getting in and out of bed, dressing, bathing and showering), domestic tasks (e.g. cleaning, ironing, cooking), meals-on-wheels (i.e. ready-made meals provided by a municipality or a private provider), and other activities (e.g. filling a drug dispenser).
  - In HRS, help received for each ADL was assessed with a question such as: “Because of a health or memory problem did anyone help you with dressing, including putting on shoes and socks in the last three months of your life?” with two possible responses: yes or no.

The present study simply used a binary indicator for receipt of ADL assistance: yes (including formal and informal, paid and non-paid) versus no.

**Formula to calculate the (Erreygers' corrected) concentration index**

To measure inequality in receipt of assistance with ADLs over the socioeconomic distribution, we calculated the concentration index (one of those recommended by World Bank to measure socioeconomic inequality in the health sector) for each survey wave in each country^1^. Briefly, the concentration index is twice the covariance between a binary indicator (here, receipt of ADL assistance) and the person’s fractional rank in the country-specific distribution of the wealth index, divided by the country-specific mean rate of (in this case) receipt of ADL assistance^1^. Theoretically, the value of the concentration index is within −1 to 1. If ADL assistance is not correlated with an individual’s position in the wealth distribution, the value of the concentration index is zero, indicating no socioeconomic inequality (though note the difference between this and equity, as discussed in the main text). A positive concentration index indicates means a disproportionate concentration of ADL assistance towards richer individuals (namely “pro-rich”), and conversely for a negative index (“pro-poor”).

To understand whether help was given to people with greater difficulties with ADLs, besides the wealth-referenced concentration index described above, we also calculated the difficulty-referenced concentration index, by replacing wealth with the extent of difficulty as the reference variable. Positive values indicate help being provided more to those with greater difficulties (“pro-difficulty”) and negative values the converse (“anti-difficulty”).

Kakwani gave a regression-based model to calculate the concentration index^2^:

$$2\sigma_{R}^{2}\frac{y_{i}}{\overline{y}}=\gamma_{1}+\delta R_{i}+\varepsilon_{i}$$

where $y_{i}$ is the status of receipt of ADL assistance, $\overline{y}$ is the mean rate of $y_{i}$, $R_{i}$ is the fractional rank of income or extent of ADL difficulty, $\sigma_{R}^{2}$is the variance of $R_{i}$, δ is the value of concentration index, and $\varepsilon_{i}$ is the error term. The advantage of this regression-based model is that it enables calculation of the 95% confidence interval of the concentration index, and calculation of a standardised concentration index by controlling other covariates. In the current study, we adopt Kakwani’s method^1^. The wealth-referenced concentration index was standardised for differences in age, sex, and the extent of ADL difficulty; and the difficulty-referenced concentration index was standardised for differences in age, sex, and wealth.

However, when the concentration index method is applied to a binary variable, the bounds of the concentration Index depend upon the rate of ADL assistance and hence make a comparison of countries with different rates of ADL assistance problematic^3^. Further, the ordering of countries by degree of inequality can depend on whether the concentration index is used to measure inequality in “receipt” versus “lack of receipt” of ADL assistance^4^. We avoided these limitations by using Erreygers' corrected concentration index^5^:

$$Erreygers^{'}correctedconcentrationindex=\frac{4\times\overline{y}}{max\left( y_{i} \right)-min\left( y_{i} \right)}\times concentrationinde$$

1. World Bank. Analyzing Health Equity Using Household Survey Data: A Guide to Techniques and Their Implementation: World Bank, 2007.

2. Kakwani N, Wagstaff A, vanDoorslaer E. Socioeconomic inequalities in health: Measurement, computation, and statistical inference. *J Econometrics* 1997; **77**(1): 87-103.

3. Wagstaff A. The bounds of the concentration index when the variable of interest is binary, with an application to immunization inequality. *Health Econ* 2005; **14**(4): 429-32.

4. Clarke PM, Gerdtham UG, Johannesson M, Bingefors K, Smith L. On the measurement of relative and absolute income-related health inequality. *Social science & medicine (1982)* 2002; **55**(11): 1923-8.

5. Erreygers G. Correcting the concentration index. *Journal of health economics* 2009; **28**(2): 504-15.

**Measures of wealth status**

The wealth measure in ESLA and HRS is a combined measure. The questionnaire contains hundreds of questions regarding respondents’ economic situation (earnings, benefits, pensions, assets, debts, etc) and they are spread over several different modules: Work and Pensions (WP), Income and Assets (IA) and Housing (HO).

The wealth measure in SHARE is level of income.

**SUPPLEMENTARY TABLE 1. Testing the influence of pandemic on the association between national level factors and outcomes of interest. To test the effect of the COVID-19 pandemic on the association between national-level factors and outcomes of interest, we fitted linear regression models, using outcomes (shown in columns) as the dependent variable. Predictor variables were the relevant national-level factor (shown in rows), COVID-19 pandemic (yes = data collected in the year 2020; no = collected earlier), and the interaction term, national-level factor × COVID-19 pandemic. The table shows the effects of the interaction term in each case.**

|  | **Percentage of people receiving assistance with ADLs** | | **Wealth equality/inequality in receiving assistance with ADLs** | | **Needs-based equity/inequity in receiving assistance with ADLs** | |
| --- | --- | --- | --- | --- | --- | --- |
|  | **Coefficient (95%CI)** | **P** | **Coefficient (95%CI)** | **P** | **Coefficient (95%CI)** | **P** |
| **A: Gross national income per capita (1000 US dollars)** | **-0.900(-1.338, -0.462)** | **0.0001** | **0.003(0.001, 0.005)** | **0.0008** | **-0.004(-0.007, 0.000)** | **0.0388** |
| **B: Active ageing index** | **-3.876(-5.698, -2.054)** | **0.0001** | **0.012(0.005, 0.020)** | **0.0017** | **-0.016(-0.028, -0.003)** | **0.016** |
| **C: Public expenditure on health (% of GDP)** | **-5.539(-9.313, -1.766)** | **0.0048** | 0.008(-0.009, 0.024) | 0.3586 | -0.015(-0.043, 0.012) | 0.262 |
| **D: Formal LTC workers, Personal carers per 100 population aged 65 years old and over** | -4.145(-8.716, 0.425) | 0.0733 | **0.020(0.001, 0.040)** | **0.0448** | -0.033(-0.076, 0.009) | 0.1174 |
| **E: Formal LTC workers, Nurses per 100 population aged 65 years old and over** | -1.189(-13.018, 10.640) | 0.8351 | 0.024(-0.040, 0.088) | 0.4425 | 0.003(-0.125, 0.131) | 0.9587 |
| **F: Percentage of population volunteering to provide ADL support** | **-1.498(-2.413, -0.583)** | **0.0018** | **0.005(0.001, 0.009)** | **0.011** | -0.005(-0.012, 0.002) | 0.1537 |

**SUPPLEMENTARY FIGURE 1. Sensitivity analysis. Percentage of people receiving assistance with ADLs plotted against national-level health system factors, including only including data from SHARE. Conventions as for Figure 3.**

**
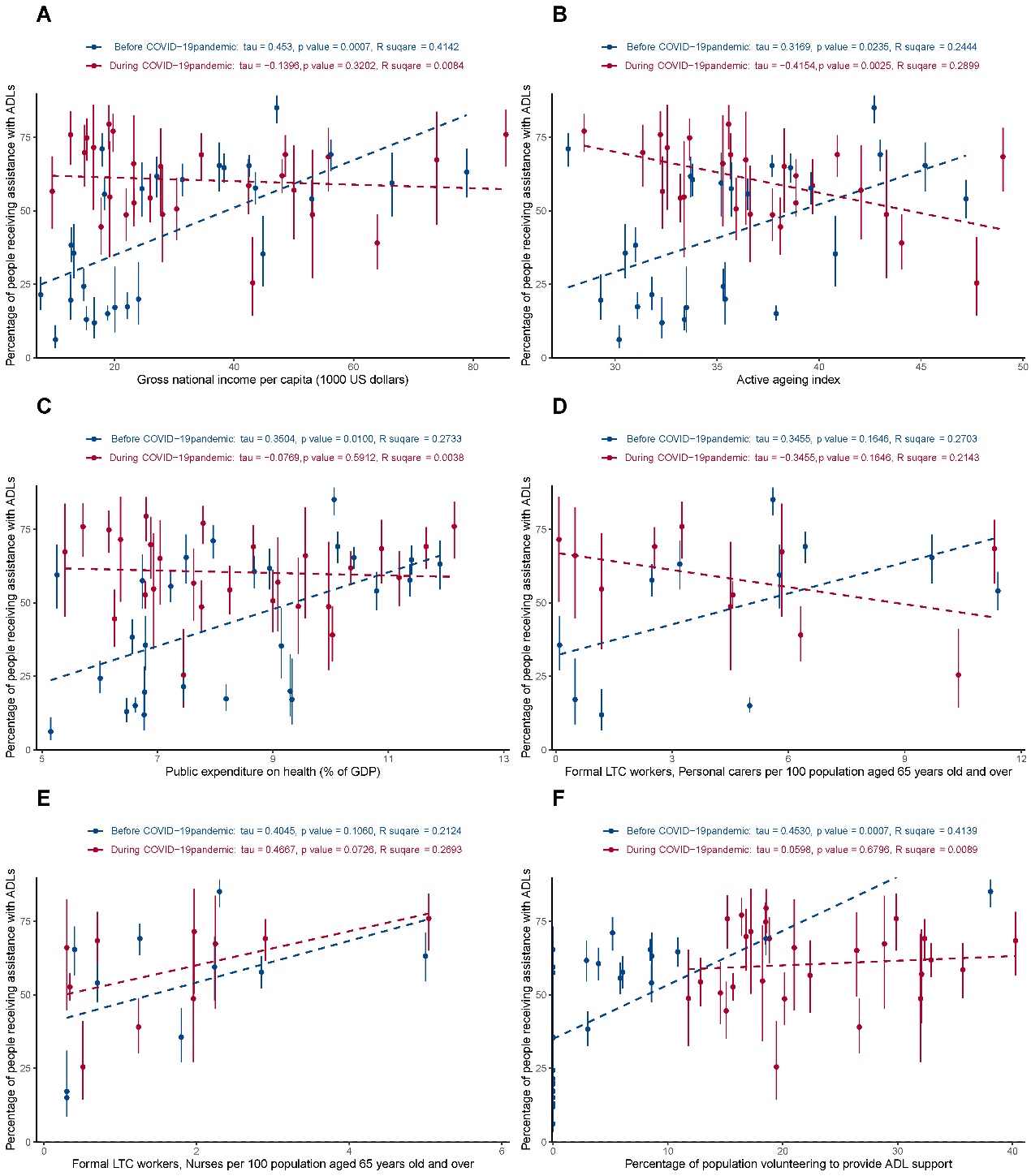
**

**SUPPLEMENTARY FIGURE 2. Sensitivity analysis. Wealth equality/inequality in receiving assistance with ADLs plotted against national-level health system factors, including only data from SHARE. Conventions as for Figure 4.**


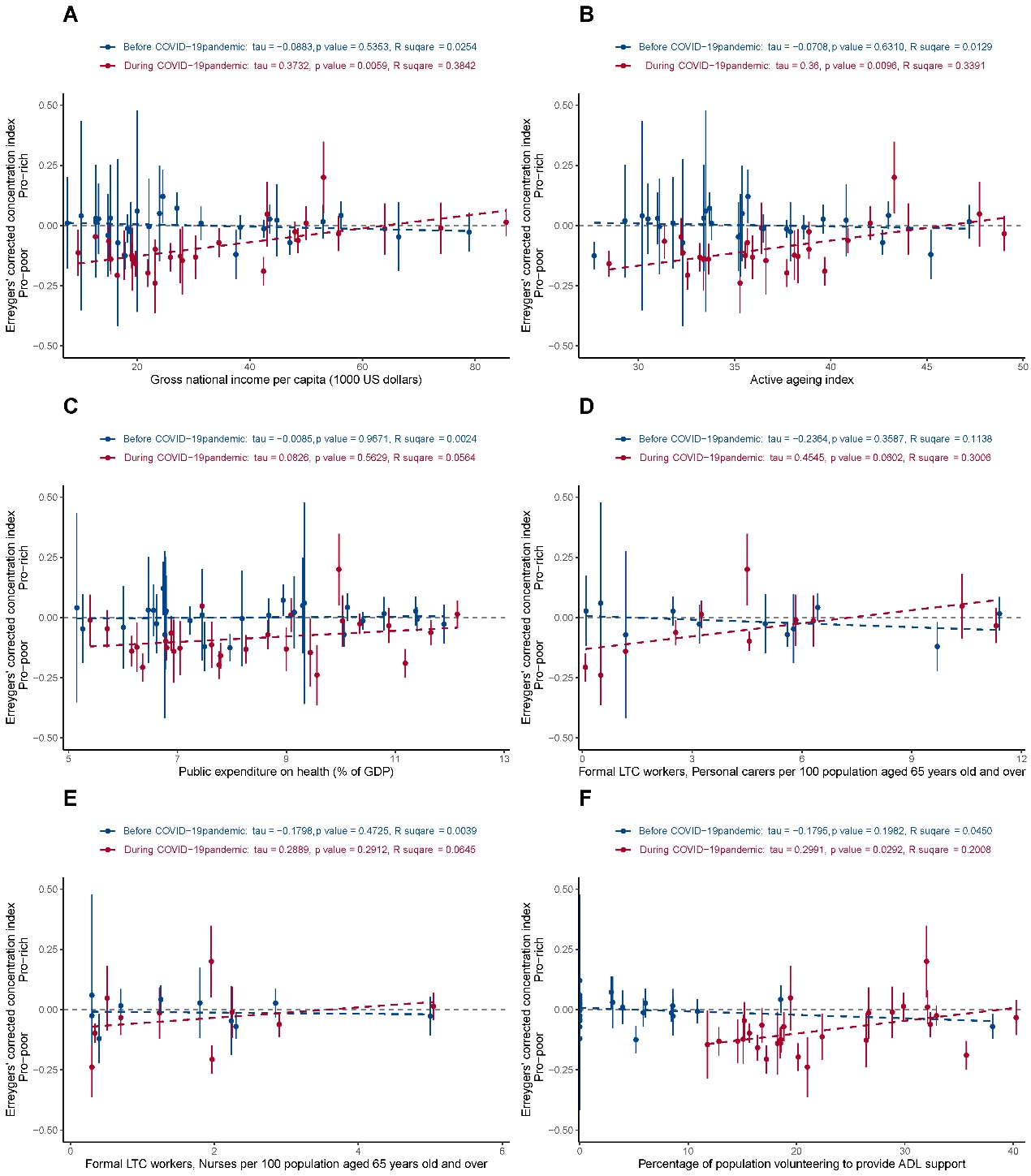


**SUPPLEMENTARY FIGURE 3. Sensitivity analysis. Needs-based equity/inequity in receiving assistance with ADLs plotted against national-level health system factors, including only data from SHARE. Conventions as for Figure 5.**

**
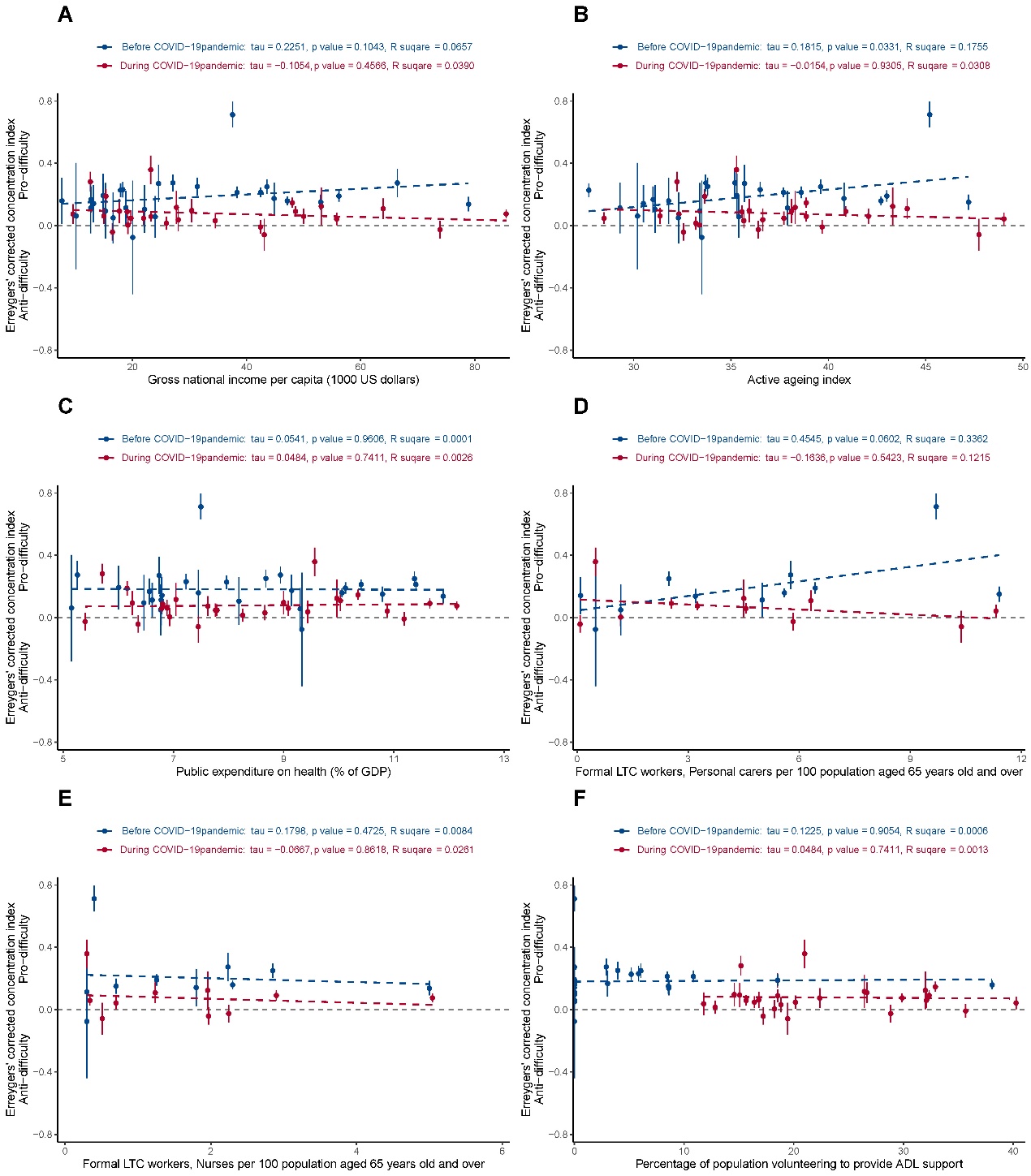
**

**SUPPLEMENTARY FIGURE 4. Change on the percentage of population volunteering to provide ADL support against Change on the percentage of people receiving assistance with ADLs.** For countries with a near-zero percentage of population volunteering to provide ADL support before pandemic (red points), an increase in the percentage of the population volunteering was more likely to be associated with an increased percentage of people receiving assistance with ADLs (red points mainly located in first quadrant). For countries that already had a substantial volunteer ADL workforce before pandemic (blue points), an increase of the percentage volunteering may not be associated with an increased percentage of people receiving assistance with ADLs (blue points located in both the first and fourth quadrants).


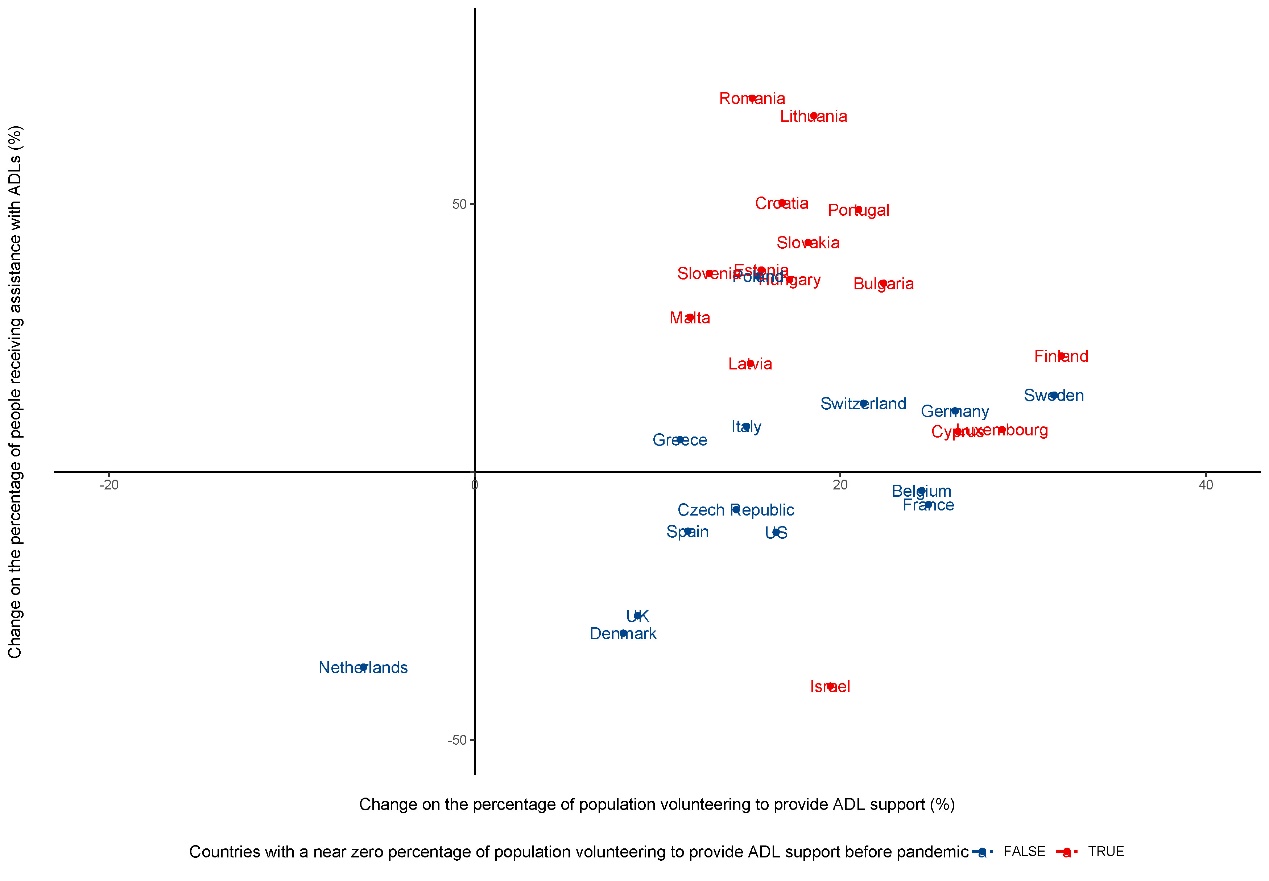

Supplement: Supplementary file 1 — Additional file 1: Supplementary Methods. Questions used in data collection, formula to calculate the (Erreygers’ corrected) concentration index, and measures of wealth status. Supplementary Table 1. Testing the influence of the COVID-19 pandemic on the association between national level factors and outcomes of interest. Supplementary Figures 1-3. Sensitivity analyses, with conventions as for Figs. 3, 4, 5, respectively. Supplementary Figure 4. Change in the percentage of population volunteering to provide ADL support against change in the percentage of people receiving assistance with ADLs. [file 12877_2022_2799_MOESM1_ESM.docx]
